# Supplementary material for: Birds migrate longitudinally in response to the resultant Asian monsoons of the Qinghai-Tibet Plateau uplift
Source: eLife. 2025 Nov 5;14:RP103971. doi: 10.7554/eLife.103971 (PMC12588604; doi:10.7554/eLife.103971)
Supplement: Supplementary file 1. [file elife-103971-supp1.docx]

|  | **Species** | **Latin** | **AUC (Breeding)** | **AUC (Wintering)** |
| --- | --- | --- | --- | --- |
| **1** | Amur Falcon | *Falco amurensis* | 0.9887 | 0.9915 |
| **2** | Bar-headed Goose | *Anser indicus* | 0.9097 | 0.9277 |
| **3** | Barn Swallow | *Hirundo rustica* | 0.962 | 0.97 |
| **4** | Bluethroat | *Cyanecula svecica* | 0.9741 | 0.9627 |
| **5** | Booted Eagle | *Hieraaetus pennatus* | 0.9693 | 0.9757 |
| **6** | Brown Shrike | *Lanius cristatus* | 0.9704 | 0.9634 |
| **7** | Citrine Wagtail | *Motacilla citreola* | 0.9538 | 0.9669 |
| **8** | Common Crane | *Grus grus* | 0.9951 | 0.9755 |
| **9** | **Common Cuckoo*** | *Cuculus canorus* | 0.9831 | 0.9643 |
| **10** | Common Greenshank | *Tringa nebularia* | 0.9212 | 0.9228 |
| **11** | Common Redshank | *Tringa totanus* | 0.9241 | 0.9476 |
| **12** | Common Ringed Plover | *Charadrius hiaticula* | 0.9845 | 0.967 |
| **13** | Common Rosefinch | *Carpodacus erythrinus* | 0.9543 | 0.9775 |
| **14** | Common Snipe | *Gallinago gallinago* | 0.992 | 0.9404 |
| **15** | **Common Swift*** | *Apus apus* | 0.9578 | 0.9723 |
| **16** | Curlew Sandpiper | *Calidris ferruginea* | 0.9633 | 0.9782 |
| **17** | **Demoiselle Crane*** | *Anthropoides virgo* | 0.9846 | 0.9871 |
| **18** | Dunlin | *Calidris alpina* | 0.9852 | 0.9394 |
| **19** | Eurasian Hobby | *Falco subbuteo* | 0.9584 | 0.9934 |
| **20** | **Eurasian Nightjar*** | *Caprimulgus europaeus* | 0.9802 | 0.9888 |
| **21** | Eurasian Whimbrel | *Numenius phaeopus* | 0.9528 | 0.9347 |
| **22** | Eurasian Wryneck | *Jynx torquilla* | 0.9625 | 0.9529 |
| **23** | Eurasian Wigeon | *Mareca penelope* | 0.9704 | 0.9551 |
| **24** | Gadwall | *Mareca strepera* | 0.9691 | 0.9482 |
| **25** | Greater Short-toed Lark | *Calandrella brachydactyla* | 0.9574 | 0.983 |
| **26** | Greater Spotted Eagle | *Clanga clanga* | 0.9431 | 0.9281 |
| **27** | Greenish Warbler | *Phylloscopus trochiloides* | 0.9548 | 0.9769 |
| **28** | Kentish Plover | *Charadrius alexandrinus* | 0.9388 | 0.9417 |
| **29** | Lesser Kestrel | *Falco naumanni* | 0.9642 | 0.9102 |
| **30** | Little Bunting | *Emberiza pusilla* | 0.9951 | 0.9614 |
| **31** | Little Ringed Plover | *Charadrius dubius* | 0.9248 | 0.9477 |
| **32** | Little Stint | *Calidris minuta* | 0.9647 | 0.9611 |
| **33** | Long-legged Buzzard | *Buteo rufinus* | 0.973 | 0.9744 |
| **34** | Marsh Sandpiper | *Tringa stagnatilis* | 0.9404 | 0.9388 |
| **35** | Northern Lapwing | *Vanellus vanellus* | 0.9791 | 0.9358 |
| **36** | Northern Shoveler | *Spatula clypeata* | 0.9544 | 0.9419 |
| **37** | **Northern Wheatear*** | *Oenanthe oenanthe* | 0.979 | 0.9853 |
| **38** | **Pallid Harrier*** | *Circus macrourus* | 0.9928 | 0.9838 |
| **39** | Pied Avocet | *Recurvirostra avosetta* | 0.9571 | 0.961 |
| **40** | Richard's pipit | *Anthus richardi* | 0.9113 | 0.9356 |
| **41** | Ruff | *Calidris pugnax* | 0.982 | 0.958 |
| **42** | Short-toed Snake-Eagle | *Circaetus gallicus* | 0.9707 | 0.9857 |
| **43** | Spotted Redshank | *Tringa erythropus* | 0.9675 | 0.9688 |
| **44** | Steppe Eagle | *Aquila nipalensis* | 0.9973 | 0.9648 |
| **45** | Terek Sandpiper | *Xenus cinereus* | 0.9626 | 0.9737 |
| **46** | Tree Pipit | *Anthus trivialis* | 0.9774 | 0.9752 |
| **47** | Western Yellow Wagtail | *Motacilla flava* | 0.9609 | 0.9536 |
| **48** | **Willow Warbler*** | *Phylloscopus trochilus* | 0.9768 | 0.9808 |
| **49** | Wood Sandpiper | *Tringa glareola* | 0.9256 | 0.9283 |
| **50** | Yellow-breasted Bunting | *Emberiza aureola* | 0.9959 | 0.9969 |

* Tracking data are available.
